# Supplementary material for: Influence of Debaryomyces hansenii on bacterial lactase gene diversity in intestinal mucosa of mice with antibiotic-associated diarrhea
Source: PLoS One. 2019 Dec 6;14(12):e0225802. doi: 10.1371/journal.pone.0225802 (PMC6897403; doi:10.1371/journal.pone.0225802)
Supplement: Attachment — Submitted filename: Response to Reviewers.docx [file pone.0225802.s004.docx]

Dear editor and reviewers:

Firstly, thanks reviewers and editor for their valuable comments on our manuscript entitled " Influence of *Debaryomyces hansenii* on bacteria lactase gene diversity in intestinal mucosa of mice with antibiotic-associated diarrhea" (PONE-D-19-19505). Those comments are very helpful for revising and improving our paper, as well as the important guiding significance to other research. We have studied the comments carefully and made corrections which we hope meet with approval. The main corrections are in the manuscript and the response to the reviewers’ comments are as follows: 
Replies to the reviewers’ comments:

**Reviewer #1:**
1.The antibiotic-associated diarrhea (AAD) mice model is the core and principal content of this manuscript. However, there is no evidence that can comfirm the model is sucsessfully established. Please provide the data.

**Response:** The antibiotic-associated diarrhea (AAD) first established by Ao Zeng, on the basis of which our research group has done a lot of researches, including the diversity of intestinal contents and intestinal mucosal bacteria, and the diversity of intestinal contents and intestinal mucosal lactase gene, fully demonstrating the reliability of this model.

1. Zeng A, Zhang HL, Tan ZJ, et al. The construction of mice diarrhea model due to dysbacteriosis and curative effect of ultra-micro Qiweibaizhusan. Microbiology China. 2012; 39(9): 1341-1348. https://doi.org/ 10.13344/j.microbiol.china.2012.09.012

2. Long C, Liu Y, He L, et al. Bacterial lactase genes diversity in intestinal mucosa of mice with dysbacterial diarrhea induced by antibiotics. 3 Biotech. 2018; 8(3): 176. https://doi.org/ 10.1007/s13205-018-1191-5 PMID: 29556430

3. Long CX, He L, Guo YF, et al. Diversity of bacterial lactase genes in intestinal contents of mice with antibiotics-induced diarrhea. World Journal of Gastroenterology. 2017; 23(42):7584-7593. https://doi.org/ 10.3748/wjg.v23.i42.7584 PMID: 29204058

2. As the authors mentioned “There are about 1000 species of bacteria inhabiting in human’s intestinal tract”, while they checked the β-galactosidase gene only in *Lactobacillus* and *Escherichia coli*. Why choose these two? Can the β-galactosidase genes in *Lactobacillus* and *Escherichia coli* represent that in all intestinal microorganisms?

**Response:** There are few lactase gene sequences published in NCBI database. We downloaded published 89 bacterial lactase gene sequences from NCBI database, and the sequence sources of these genes include *Bifidobacterium*, *Lactobacillus*, *Enterobacter*, *Streptococcus*, *Bacillus*, *Micrococcus* and *Clostridium.* According to these bacterial lactase gene sequences, we designed 7 pairs of universal primers for the analysis of bacterial lactase gene diversity. After PCR amplification and metagenomic sequencing, it was found that universal primers of bacterial lactase gene from *Lactobacillus* and *Escherichia coli* had good specificity, high sensitivity and could annotate more microbial species.

1. Long CX, He L, Liu YJ, et al. Universal primer for analysis of the diversity of intestinal bacterial lactase gene. Chinese Journal of Applied and Environmental Biology. 2017, 23(04): 758-763. https://doi.org/ 10.3724/SP.J.1145.2016.10008

3. Eighteen mice, which were randomly divided into three groups (6 mice per group), healthy control group, diarrhea model group and *D. hansenii* treatment group, were used in the present study. However, they used samples from nine mice for β-galactosidase gene amplification and sequencing. Why not check the β-galactosidase gene in all eighteen mice? Do you think the data from three sample per group is enough for clarification the influence of *Debaryomyces hansenii* on bacteria lactase gene diversity in intestinal mucosa of mice with antibiotic-associated diarrhea?

**Response:** Admittedly, the bioduplication of the three samples in each group is a little low, but it can meet the minimum requirements of statistics. In our experiment, there were 6 mice in each group. When collecting intestinal mucosa, we randomly mixed the mucosa of a male and a female mouse in the group. On the one hand, the intestinal mucosa of mice was small, and we were worried that the extracted DNA was not pure enough. On the other hand, there were some differences between the intestinal flora of male mice and female mice. In order to fully reflect the characteristics of the mucosal bacteria of mice, the intestinal mucosa of a male mouse and a female mouse were mixed together.

4. The writing need to be improved.
**Response:** Thanks for the expert's advice. We will check the manuscript carefully to improve the readability of the article.

**Reviewer #2:**

1. Throughout the manuscript, the use of the English language needs to be drastically improved. Numerous grammatical errors are present in the manuscript. This is a major concern that must be addressed before publication.

**Response:** Thanks to the expert's valuable advice, we will ask a native English speaker to polish our manuscript.

2. More references are appropriate, such as for the experimental techniques. For example, what is the source reference for the universal primers according to lactase gene sequences of *Lactobacillus* and *Escherichia coli* ?

**Response:** The sources of the universal primers for the analysis of lactase gene diversity were designed by our research group. According to the lactase gene sequence registered in NCBI database, DANMAN software was used to find the conservative region of the lactase gene sequence, and the Primer Premier 5.0 software was used to design upstream and downstream primers on both ends of conserved regions. Finally, the synthesis of primers was completed by Shanghai pessenol biotechnology co., LTD.

1. Long CX, He L, Liu YJ, et al. Universal primer for analysis of the diversity of intestinal bacterial lactase gene. Chinese Journal of Applied and Environmental Biology. 2017, 23(4): 758-763. https://doi.org/ 10.3724/SP.J.1145.2016.10008
2. The author should provide the data about the fecal consistency, fecal water content and stool weight after treatment with antibiotics or *D. hansenii* ?

**Response:** In this study, we did not conduct quantitative analysis of fecal concentration, fecal water content and fecal weight after treatment with antibiotics or *D. hansenii*, but in previous articles, we have qualitatively recorded the wetness of stool after antibiotic modeling and yeast treatment in mice.

1. Guo KX, Tan ZJ, Xie MZ, et al. The synergic effect of ultra-micro powder Qiweibaizhusan combined with yeast on dysbacteriotic diarrhea mice. Chinese Journal of Applied and Environmental Biology. 2015; 21(1): 61-67. https://doi.org/ 10.3724/sp.j.1145.2013.10002

Replies to the editors’ suggestions

1. You should revise your language with a native English speaker.

**Response:** Thanks for the editor's advice, in terms of language quality, we invited our colleagues working in the United States to polish the manuscript to make it more accurate.

2. You should upload your raw data into public database such as NCBI and provide the accession number. Table 1 should be deleted as the data could be gained according to your raw data.

**Response:** As required, we have uploaded all the original sequences to NCBI database. The accession number is SRP220043 (https://www.ncbi.nlm.nih.gov/Traces/sra_sub/), which was released on September 6, 2019. According some information on the Internet, the data uploaded to NCBI database might not be retrieved for 3 months. So I listed my NCBI database(https://www.ncbi.nlm.nih.gov/) account and password at the end. **Account number**: heyunshan; **password**: 20183371he

3. For data analysis, you should perform data normalization, i.e. each sample with same read numbers, before the subsequent data analysis.

**Response:** Thanks to the editors for their advice, the sample size of each group is consistent in any one data analysis. The data analysis of this experiment was conducted by professionals of pessenol biological company, and the data were authentic and reliable with strong comparability.

4. Alpha diversity analysis should included richness indices and diversity indices. Please added these indices.

**Response:** Regarding Alpha diversity, Simpson index was added, which could well reflect community diversity.

5. For PCoA analysis, you should include different algorithms such as weighted UniFrac, unweighted UniFrac and Bray-Curtis.

**Response:** For beta analysis, PCA and NMDS analysis are selected instead of PCoA analysis. NMDS analysis is not affected by the numerical value of sample distance, and only considers the size relationship between them. For data with complex structure, NMDS analysis ranking results may be more stable.

6. For differential bacteria analysis, you should perform LEfSe analysis.

**Response:** In the differential bacteria analysis, we introduced LEfS analysis as required. LEfSe analysis show that *Sphingomonadaceae*, *Sphingomonas* and *Sphingomonadales* were the key colony members in *D. hansenii* treatment group, while no key colony members were found in healthy control group and diarrhea model group.
